# Results of the global conservation assessment of the freshwater crabs (Brachyura, Pseudothelphusidae and Trichodactylidae): The Neotropical region, with an update on diversity

**DOI:** 10.3897/zookeys.457.6598

**Published:** 2014-11-25

**Authors:** Neil Cumberlidge, Fernando Alvarez, Jose-Luis Villalobos

**Affiliations:** 1 Department of Biology, Northern Michigan University, Marquette, MI 49855, USA; 2 Colección Nacional de Crustáceos, Instituto de Biología, Universidad Nacional Autónoma de México, Apartado Postal 70–153, México 04510, D. F. México

**Keywords:** Pseudothelphusidae, Trichodactylidae, Neotropical region, conservation, distribution, endemism

## Abstract

The freshwater crabs of the Neotropics comprise 311 species in two families (Pseudothelphusidae and Trichodactylidae) and one or both of these families are found in all of the countries in the Neotropical region (except for Chile and some of the Caribbean islands). Colombia (102 species, 81% endemic) and Mexico (67 species, 95% endemic) are the biodiversity hotspots of freshwater crab species richness and country-level endemism for this region. The results of the IUCN Red List conservation assessments show that 34% of pseudothelphusids and 10% of trichodactylids have an elevated risk of extinction, 29% of pseudothelphusids and 75% of trichodactylids are not at-risk (Least Concern), and although none are actually extinct, 56% of pseudothelphusids and 17% of trichodactylids are too poorly known to assess (Data Deficient). Colombia (14 species), Venezuela (7 species), Mexico (6 species), and Ecuador (5 species) are the countries with the highest number of threatened species of Neotropical freshwater crabs. The majority of threatened species are restricted-range semiterrestrial endemics living in habitats subjected to deforestation, alteration of drainage patterns, and pollution. This underlines the need to prioritize and develop conservation measures before species decline to levels from which they cannot recover. These results represent a baseline that can be used to design strategies to save threatened Neotropical species of freshwater crabs.

## Introduction

The Neotropical region occupies the entire South American continent, plus Mexico and Central America, the islands of the Caribbean, and southern Florida. The Nearctic/Neotropical boundary runs through Mexico and passes through the southern parts of Baja California and Sonora, crosses the Meseta Central, and continues to southern Veracruz. The majority of the Neotropical region has a tropical climate with warm water freshwater ecosystems, but the southern part of this region from 10° to 25°S (southern Brazil, Uruguay, Paraguay, Argentina, and Chile) has a subtropical climate with cooler freshwater habitats. Freshwater crabs are found throughout the freshwater ecosystems of the Neotropical region but are notably absent from Baja California and the Yucatan Peninsula (Mexico), southern Florida (USA), some of the Caribbean islands, southern Argentina, and all of Chile.

Our knowledge of the impressive freshwater crab fauna in this region has been very slow to develop since the first species of trichodactylid and pseudothelphusid freshwater crabs were described in 1783 and 1840, respectively. Only one species (a trichodactylid) was described in the 18^th^ C (Herbst 1783), 46 more species were described in the 19^th^ C (Ortmann 1893, 1897; Rathbun 1893, 1898), 209 more species were added in the 20^th^ C (Rathbun 1905, Smalley 1964a, b, 1965, 1970, Bott 1967, 1968, 1969, 1970, Rodríguez and Smalley 1969, Pretzmann 1972, 1975, Hobbs 1980, Rodríguez 1980, 1982, Campos and Rodríguez 1984, 1985, 1993, 1995, Smalley and Adkison 1984, Magalhães 1986, Magalhães and Türkay 1986, Rodríguez 1986, Alvarez 1987, 1989, Campos and Rodríguez 1988, Campos 1989, Alvarez and Villalobos 1990, 1991, 1994, 1995, 1996, 1997a,b, 1998, Hobbs 1991, Campos 1992, 1999, Rodríguez 1992, Villalobos et al. 1993, Campos 1995, 1997, Alvarez et al. 1996, 1999, Magalhães and Türkay 1996a, b, c, Campos and Lemaitre 1998), and 31 species have been described so far in the 21^st^ C (Campos 2000, 2001, 2002, 2003a, b, 2004, 2005, 2010a, b, 2011, Campos and Lemaitre 2002, Rodríguez et al. 2002, Villalobos and Alvarez 2003, 2009, 2010, 2013, Campos and Valencia 2004, Rodríguez and Magalhães 2005, Villalobos 2005, Campos and Pedraza 2006, 2008, Cumberlidge 2007, Cumberlidge et al. 2009, Magalhães and Türkay 2008a, b, c, 2012, Ng et al. 2008, Yeo et al. 2008, Magalhães 2010, Magalhães et al. 2010, Magalhães and Türkay 2010, Villalobos et al. 2010, Alvarez et al. 2012a, Cardona and Campos 2012, Magalhães et al. 2013). By 2009, the total number of Neotropical species stood at more than 300, making this the second most species-rich region in the world after the Oriental region (Cumberlidge et al. 2009, Ng et al. 2008). Interest in the Neotropical freshwater crab fauna remains strong and 13 new species have already been recognized since 2009 (Table 1). Exploration is still continuing and there is every prospect that the species count will increase further as taxonomic discrimination improves. We expect most of the new species to belong to the Pseudothelphusidae, because discovery of new species of trichodactylids has slowed dramatically in the last two decades.

**Table 1.** Species of Neotropical freshwater crabs described since Cumberlidge et al. (2009).

Family	Subfamily/Tribe	Species	Country
Pseudothelphusidae	Kingsleyini	*Microthelphusa lipkei* Magalhães, 2010	Brazil
Pseudothelphusidae	Kingsleyini	*Brasiliothelphusa dardanelosensis* Magalhães & Türkay, 2010	Brazil
Pseudothelphusidae	Strengerianini	*Phallangothelphusa juansei* Campos, 2010	Colombia
Pseudothelphusidae	Strengerianini	*Phallangothelphusa martensis* Cardona & Campos, 2012	Colombia
Pseudothelphusidae	Hypolobocerini	*Neostrengeria alexae* Campos, 2010	Colombia
Pseudothelphusidae	Hypolobocerini	*Neostrengeria natashae* Campos, 2011	Colombia
Pseudothelphusidae	Potamocarcinini	*Potamocarcinus darienensis* Magalhães, Campos & Türkay, 2013	Panama
Pseudothelphusidae	Hypolobocerini	*Allacanthos yawi* Magalhães, Lara & Wehrtmann, 2010	Costa Rica
Pseudothelphusidae	Potamocarcinini	*Odontothelphusa apicpac* Villalobos, García & Velázquez, 2010	Mexico
Pseudothelphusidae	Potamocarcinini	*Sylvathelphusa kalebi* Villalobos & Alvarez, 2013	Mexico
Pseudothelphusidae	Potamocarcinini	*Sylvathelphusa cavernicola* Villalobos & Alvarez, 2013	Mexico
Pseudothelphusidae	Potamocarcinini	*Tzotzithelphusa villarosalensis* Villalobos & Alvarez, 2013	Mexico
Pseudothelphusidae	Pseudothelphusini	*Pseudothelphusa zongolicae* Alvarez, Villalobos & Moreno, 2012	Mexico

Research on the Neotropical freshwater crabs has also focused on their phylogeny (Rodríguez and Campos 1989, 1998, Rodríguez and Pereira 1992, Rodríguez and Sternberg 1998, Sternberg et al. 1999, Sternberg and Cumberlidge 2001, Rodríguez and Magalhães 2005, Campos 2005, Huidobro et al. 2006, Villalobos and Alvarez 2010), their biogeography (Rodríguez 1986, Alvarez et al. 2012b, Ojeda et al. 2013), and their medical importance with 22 species of pseudothelphusids serving as second intermediate hosts of the human lung fluke, *Paragonimus* (Acha and Szyfres 2001) in 9 countries from Mexico and Central America, to the Andes, and Brazil (Rodríguez and Magalhães 2005).

The objectives of the present study are to describe and update our knowledge of Neotropical freshwater crab diversity of both the Pseudothelphusidae and Trichodactylidae and to describe the patterns of distribution and endemism within these families. We also identify here not only those species that are most vulnerable to extinction, but also those species that are poorly known and obvious candidates for future research attention.

## Methods

**Figure 1.**
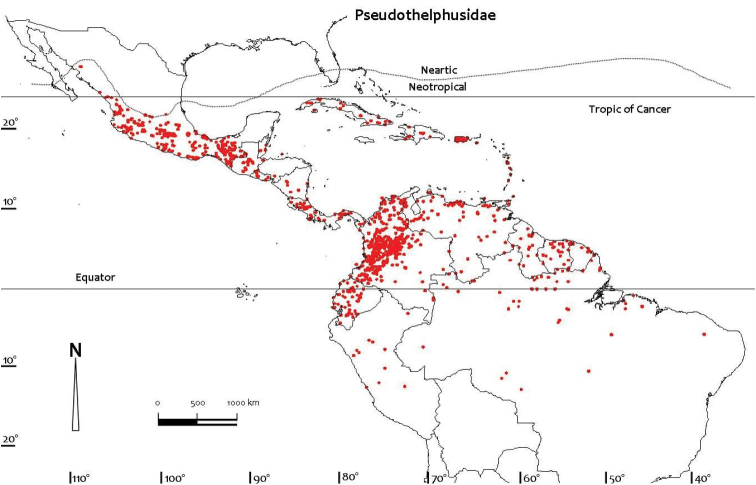
Distribution of Pseudothelphusidae based on all known point localities (n = 1719).

**Figure 2.**
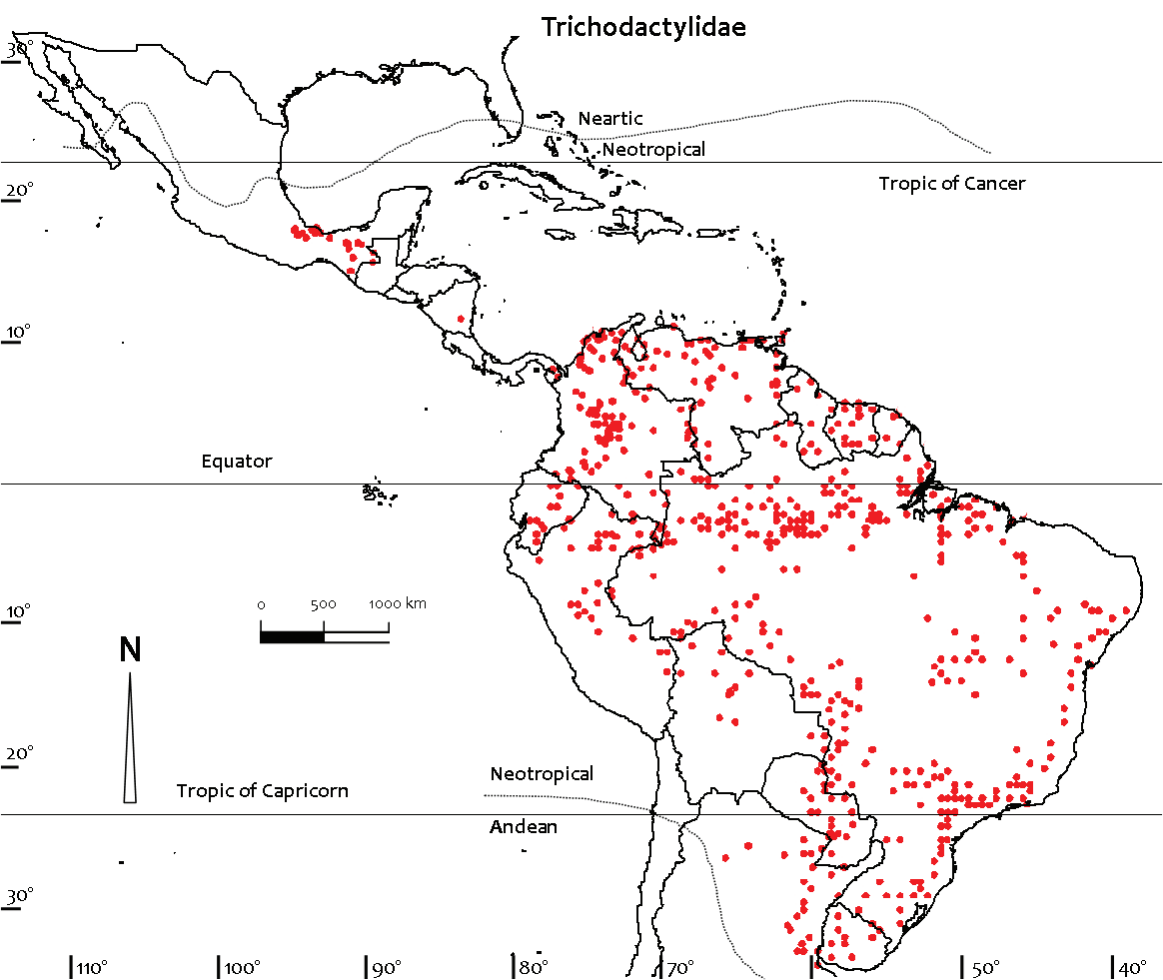
Distribution of Trichodactylidae based on all known point localities (n = 853).

**Figure 3.**
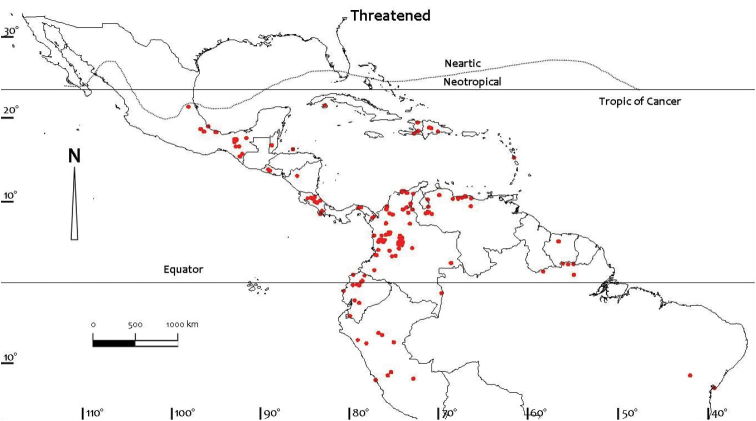
Distribution of threatened species of Neotropical freshwater crabs (Pseudothelphusidae and Trichodactylidae) based on all known point localities (n = 173).

**Figure 4.**
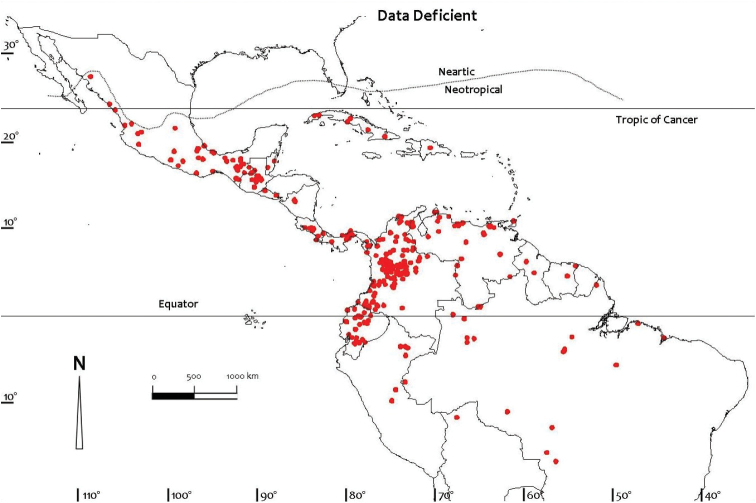
Distribution of Data Deficient species of Neotropical freshwater crabs (Pseudothelphusidae and Trichodactylidae) based on all known point localities (n = 338).

Comprehensive distributional data for the Neotropical freshwater crabs were compiled from literature and museum records, particularly the major monographs on the Pseudothelphusidae by Rodríguez (1982, 1992) and Rodríguez and Magalhães (2005), and the Trichodactylidae by Rodríguez (1992) and Magalhães and Türkay (1996a, b, c, 2008a, b, 2012). Other regional monographs referred to include those for Colombia (Campos 2005), Ecuador (Rodríguez and Sternberg 1998), Brazil (Magalhães 2003), Peru (Rodríguez and Suárez 2004), and Mexico and Central America (Alvarez and Villalobos 1990, 1991, 1994, 1995, 1996, 1997, Villalobos et al. 1993, Alvarez et al. 1996, 1999, Villalobos and Alvarez 2003, 2008, 2009, 2010, Villalobos 2005). Despite these efforts distributional records for most species of freshwater crabs are still likely to be incomplete. Distribution maps presented here are based on specimen-level databases compiled for all species based on all known specimens and includes information from over 2,500 different localities. Individual conservation assessments for all species of Neotropical freshwater crabs assessed by Cumberlidge et al. (2009) using the IUCN Red List categories and criteria at the global scale (IUCN 2003) are provided on the IUCN Red List site (http://www.iucn.redlist.org). Freshwater crab species were assessed for inclusion in one of the Red List categories based on a combination of data on geographic range and/or population levels and related trends (Cumberlidge et al. 2009). The available data were sufficient to make valid assessments of the conservation status of only 150 out of the 298 species known from the Neotropical region at the time, and assessments were not possible for 148 species that were treated as Data Deficient due to a lack of specimens, and of locality and population data (Cumberlidge et al. 2009). Threats were inferred if a species was potentially subject to anthropogenic impacts such as habitat destruction, alteration of drainage patterns, or pollution, especially if it was either not found in a protected area, or if it was found in a protected area for only part of its range. Distribution maps (Figs 1–4) were prepared using ArcView GIS mapping software.

## Results

### Neotropical freshwater crab families

The Neotropical region has its own distinctly recognizable freshwater crab fauna, with no species occurring in other parts of the world. The Neotropical freshwater crab fauna is not uniformly distributed and species and genus composition changes from country to country (Table 2, Figs 1, 2). The global distributions of all species of the Pseudothelphusidae and Trichodactylidae based on all known localities are shown in figures 1 and 2. The Neotropical freshwater crab fauna today (49 genera, 311 species, 2 families) easily exceeds that of the entire Afrotropical region (18 genera, 145 species, 2 families), but is significantly less diverse than the Palaearctic-Oriental-Australasian region (147 genera, 850 species, 2 families) (Cumberlidge et al. 2009; present work).

**Table 2.** Distribution of Pseudothelphusidae (P) and Trichodactylidae (T) in each country in the Neotropical region that has populations of freshwater crabs. First number indicates the number of genera, species, and localities of Pseudothelphusidae, the second number indicates the same data for the Trichodactylidae.

	No. GENERA (P, T)	No. SPECIES (P, T)
**South America**		
Argentina	0, 5	0, 9
Bolivia	0, 6	0, 8
Brazil	6, 10	19, 31
Colombia	13, 8	88, 14
Ecuador	3, 4	21, 5
French Guiana	2, 4	3, 4
Guyana	3, 3	8, 3
Paraguay	0, 6	0, 7
Peru	2, 9	8, 14
Suriname	3, 3	5, 3
Uruguay	0, 1	0, 2
Venezuela	11, 7	35, 9
**Mexico and Central America**		
Mexico	15, 2	63, 4
Belize	4, 0	4, 0
Costa Rica	4, 0	14, 0
El Salvador	4, 0	6, 0
Guatemala	6, 0	12, 0
Honduras	2, 0	3, 0
Nicaragua	3, 3	5, 3
Panama	4, 1	14, 1
**Caribbean**		
Cuba	2, 0	9, 0
Dominica	1, 0	1, 0
Dominican Republic	1, 0	2, 0
Guadeloupe	1, 0	1, 0
Haiti	1, 0	1, 0
Martinique	1, 0	1, 0
Puerto Rico	1, 0	1, 0
Saint Vincent & the Grenadines	1, 0	1, 0
St Croix US Virgin Islands	1, 0	1, 0
Trinidad & Tobago	2, 1	2, 1

The Neotropical freshwater crab fauna, which represent 20–22% of the world’s freshwater crab species diversity (Ng et al. 2008, Yeo et al. 2008, Cumberlidge et al. 2009), is dominated by the highly diverse pseudothelphusids (34 genera, 264 species). Pseudothelphusids are found in 26 out of the 31 countries and reach their highest species richness in South America (Table 2). Pseudothelphusids are found throughout the warmer parts of the Neotropics from Mexico to Central America and the Andes (Venezuela, Colombia, Ecuador, and Peru), and in the Guyanian Shield and Central Brazilian Shield in the Amazon basin (Fig. 1, Table 2). The majority of pseudothelphusid species live at altitudes greater than 400 m above sea level, with a few Andean species reaching up to 3000 m (Campos 2005).

This family comprises two subfamilies, Epilobocerinae and Pseudothelphusinae (Ng et al. 2008). The Epilobocerinae consists of only 9 species in 2 genera (*Epilobocera*, *Neoepilobocera*) that are all endemic to the larger islands of the Greater Antilles (Cuba, Haiti, Dominican Republic, Puerto Rico, and St. Croix). The vast majority of pseudothelphusids are in the subfamily Pseudothelphusinae assigned to five Tribes: Pseudothelphusini (Mexico), Potamocarcinini (Central America and Mexico), Hypolobocerini (from Venezuela to Peru, plus Central America, and Mexico), Strengerianini (Colombia and Venezuela), and Kingsleyini (the Lesser Antilles, Venezuela, Colombia, and northern Brazil). The eastern limits of the Pseudothelphusidae in the Amazon basin is in Ceará State, Brazil, while their southwestern limit in the Amazon basin is marked by the watershed of the Ucayali River, south of which there are no species of pseudothelphusids (Rodríguez and Magalhães 2005).

The Trichodactylidae is the smallest of all freshwater crab families (15 genera, 47 species) and represents only 4% of the world’s freshwater crab diversity. Despite this, the family is widely distributed and has representatives in 16 countries, most of which are in South America (Table 2) (Rodríguez 1992, Ng et al. 2008, Yeo et al. 2008, Cumberlidge et al. 2009). The vast majority of trichodactylid species are found below 100 m above sea level in the Magdalena river basin and Lake Maracaibo in Colombia and Venezuela, in the coastal lowlands of the Guyanas and Brazil, and in the lowland river basins of the Amazon, Orinoco, Paraguay, and Parana. A few species of trichodactylids reach up to 900 m above sea level in the Andes foothills (Campos 2005). Countries with trichodactylids include Colombia, Venezuela, French Guiana, Guyana, Suriname, Brazil, Ecuador, Peru, Bolivia, Paraguay, Uruguay, and Argentina (Fig. 2, Table 2). Outside of South America there are four trichodactylid species in Veracruz, Tabasco, Oaxaca, and Chiapas in Mexico (*Avotrichodactylus constrictus*, *Avotrichodactylus oaxensis*, *Rodriguezia mensabak*, and *Rodriguezia villalobosi*) (Magalhães and Türkay 2012); one species (*Trichodactylus quinquedentatus*) from Colombia and Nicaragua (Smalley and Rodríguez 1972), and one species (*Poppiana dentata*) from Trinidad (which is also found on the neighboring mainland in Venezuela, Suriname, Guyana, and French Guiana, and in Nicaragua) (Magalhães and Türkay 2008a). The Trichodactylidae includes two subfamilies: the Trichodactylinae, with 3 genera, two from Mexico (*Avotrichodactylus* and *Rodriguezia*) and one from South and Central America (*Trichodactylus*); and the Dilocarcininae, with 9 genera that are found throughout South America (Rodríguez 1992, Magalhães and Türkay 1996a, b, c, 2008a, b). Interestingly, this classification (Ng et al. 2008) groups together at the subfamily level the four disjunct species of trichodactylids found in Mexico with the genus *Trichodactylus* that includes species from the Magdalena basin and Amazon slopes in Colombia, a species from Nicaragua and Colombia, species from the Atlantic coastal river systems of southeastern Brazil, and species from the Paraguay and Parana basins in southern Brazil and Argentina.

### Hotspots of freshwater crab diversity

Colombia stands out as the most diverse and the most species rich part of the region (21 genera, 102 species, 2 families) (Table 2) with biodiversity hotspots located in forested mountainous regions where deep valleys and a complex topographical relief are favorable for genetic isolation and allopatric speciation. The pseudothelphusid fauna of Colombia comprises 13 genera and 88 species in one subfamily (Pseudothelphusinae) and four tribes (Hypolobocerini, Strengerianini, Kingsleyini, and Potamocarcinini). There are distinct northern and southern components of pseudothelphusid distribution in Colombia, with the dividing line coinciding with the watersheds of the San Juan, Cauca, and Magdalena river basins. The southern component of this fauna is dominated by *Hypolobocera*, *Lindacatalina*, and *Moritschus* and extends from southern Colombia into Ecuador and Peru, and also includes species from southeast Colombia and the Amazonian and Pacific slopes of Ecuador. The trichodactylid fauna of Colombia comprises 8 genera, 14 species in two subfamilies: Trichodactylinae (one genus) and Dilocarcininae (7 genera).

The next most species-rich country is Mexico (17 genera, 67 species, 2 families) (Table 2) with a freshwater crab fauna dominated by pseudothelphusids with 15 genera, and 63 species in one subfamily (Pseudothelphusinae) and three tribes (Pseudothelphusini, Potamocarcinini, and Hypolobocerini). Mexico’s trichodactylid fauna is much smaller and comprises just 4 species in 2 genera all in the subfamily Trichodactylinae. Freshwater crab diversity in Mexico is highest in the Isthmus of Tehuantepec and Chiapas State, possibly because this is where species from three pseudothelphusid tribes overlap with species of trichodactylids (Villalobos and Alvarez 2010).

Brazil is the third most species-rich Neotropical country (16 genera, 50 species, 2 families) with a fauna that is dominated by trichodactylids (10 genera, 31 species, 2 subfamilies), and fewer pseudothelphusids (6 genera, 19 species, all Pseudothelphusinae, Kingsleyini). Venezuela is the fourth most species-rich Neotropical country (18 genera, 44 species, 2 families), made up of 11 genera and 35 species of pseudothelphusids (all Pseudothelphusinae, in three tribes, Hypolobocerini, Kingsleyini, and Strengerianini), and 7 genera and 9 species of trichodactylids (all Dilocarcininae).

The freshwater crab fauna of Ecuador (7 genera, 26 species, 2 families) includes 3 genera and 21 species of pseudothelphusids (all Pseudothelphusinae, Hypolobocerini), and 4 genera and 5 species of trichodactylids (all Dilocarcininae). Finally, Peru (11 genera, 22 species, 2 families) has a fauna dominated by trichodactylids (14 species and 8 genera, in 2 subfamilies Dilocarcininae and Trichodactylinae), with a smaller pseudothelphusid fauna consisting of 8 species and 2 genera (all Pseudothelphusinae in 2 tribes Kingsleyini and Hypolobocerini). Freshwater crabs of both families are completely absent from Chile. Despite warm subtropical climates freshwater crabs are absent in many islands of the Caribbean including Jamaica, The Cayman Islands, The Bahamas, Grenada, Antigua, and Anguilla.

When distribution patterns are considered at the genus level (Table 2) the taxonomic diversity of Neotropical freshwater crabs is again by far the highest in Colombia (21 genera), Venezuela (18 genera), Mexico (17 genera), Brazil (16 genera), and Peru (11 genera). Diversity is lower in Ecuador (7 genera), Guyana, Suriname, Bolivia, Paraguay, French Guiana, Nicaragua, and Guatemala (6 genera), and in Argentina and Panama (5 genera) (Table 2). Costa Rica, El Salvador and Belize each have 4 genera, Trinidad and Tobago has 3 genera, Honduras and Cuba each have 2 genera, while Uruguay and the other islands of the Greater and Lesser Antilles each have 1 genus.

### Widespread species: Pseudothelphusidae

Thirteen (out of 34) pseudothelphusid freshwater crab genera and 28 (out of 283) species have a wide distribution that easily exceeds 20,000 km^2^ and includes more than one country. Notably, *Fredius reflexifrons* is found in six countries (Brazil, French Guiana, Guyana, Peru, Suriname, Venezuela), *Potamocarcinus magnus* is found in five countries (Mexico, Guatemala, Honduras, El Salvador, Costa Rica), and *Kingsleya latifrons* (Guyana, French Guiana, Suriname, Brazil) and *Raddaus bocourti* (Mexico, Belize, Guatemala, El Salvador) are found in four countries. Other widespread species found in three countries include *Potamocarcinus armatus* (Mexico, Guatemala, Nicaragua), *Potamocarcinus richmondi* (Nicaragua, Costa Rica, Panama), *Fredius denticulatus* (Brazil, French Guiana, Suriname) and *Fredius fittkaui* (Venezuela, Guyana, Brazil), and *Prionothelphusa eliasi* (Brazil, Colombia and Venezuela). Finally, 17 widespread species are found in two countries: *Rodriguezus garmani* (Trinidad and Tobago, Venezuela), *Fredius beccarii* (Guyana, Venezuela), *Zilchia zilchi* (El Salvador, Honduras), *Hypolobocera caputii* and *Moritschus henrici* (both Ecuador, Peru), *Ptychophallus exilipes* (Costa Rica, Panama), *Hypolobocera bouvieri angulata*, *Orthothelphusa holthuisi* (Colombia, Venezuela), and *Hypolobocera exuca*, *Lindacatalina latipenis*, *Lindacatalina orientalis*, and *Lindacatalina sumacensis* (all Colombia, Ecuador), *Fredius estevisi* and *Fredius platyacanthus* (Brazil, Venezuela), *Kingsleya siolii* (Brazil, Suriname), *Zilchia aspoekorum* (Belize, Mexico), and *Raddaus tuberculatus* (Guatemala, Mexico).

The majority of pseudothelphusids (22 species) that have a widespread distribution exceeding 20,000 km^2^ are assessed as Least Concern. However, there are some species with a small distributional range that spans the border between two neighboring countries that are assessed as either Vulnerable: *Hypolobocera exuca* (Colombia, Ecuador), *Kingsleya siolii* (Brazil, Suriname) and *Zilchia aspoekorum* (Belize, Mexico), or Data Deficient: *Fredius platyacanthus* (Brazil, Venezuela), *Lindacatalina latipenis* and *Lindacatalina sumacensis* (both Colombia, Ecuador), *Orthothelphusa holthuisi* (Colombia, Venezuela), and *Ptychophallus exilipes* (Costa Rica, Panama).

### Widespread Species: Trichodactylidae

Some 8 out of 15 trichodactylid freshwater crab genera and 20 out of 47 species have a wide distributional range exceeding 20,000 km^2^ that spans more than one country: all are assessed as Least Concern. Notably, *Valdivia serrata* is found in nine countries (Bolivia, Brazil, Colombia, Ecuador, Peru, Venezuela, Guyana, Suriname, French Guiana), *Poppiana dentata* in eight countries (Brazil, Colombia, French Guiana, Guyana, Nicaragua, Suriname, Trinidad and Tobago, Venezuela), and *Sylviocarcinus pictus* in seven countries (Colombia, Brazil, Bolivia, Peru, Argentina, French Guiana, Guyana). *Dilocarcinus pagei* is found in six countries (Argentina, Brazil, Bolivia, Colombia, Paraguay, Peru), and *Sylviocarcinus devillei* and *Moreirocarcinus emarginatus* are both found in 5 countries (Bolivia, Brazil, Colombia, Ecuador, and Peru; and Brazil, Colombia, Ecuador, Peru, and Venezuela respectively). Four species are found in four countries: *Trichodactylus kensleyi* (in Argentina, Brazil, Paraguay and Uruguay) and *Poppiana argentiniana*, *Valdivia camerani*, and *Trichodactylus borellianus* (in Argentina, Bolivia, Brazil, and Paraguay). Seven species are found in three countries (*Rotundovaldivia latidens*, *Sylviocarcinus maldonadoensis*, *Sylviocarcinus australis*, *Trichodactylus faxoni*, *Trichodactylus panoplus*, *Valdivia cururuensis* and *Zilchiopsis oronensis*), four species are found in two countries (*Bottiella niceforei*, *Bottiella cucutensis*, *Forsteria venezuelensis*, *Trichodactylus quinquedentatus*), and three species are found in one country (*Trichodactylus petropolitanus*, *Valdivia novemdentata*, and *Zilchiopsis collastinensis*).

### Distribution patterns and habitat

Freshwater crabs are found in all major habitat types in the Neotropics (Thieme et al. 2005; Abell et al. 2008) including rivers, rapids, swamps, lakes, caves, and mountain streams and species belonging to both families prefer warm-water habitats year round. The Pseudothelphusidae are typically found in small mountain streams above 400 m above sea level, which may in part reflect their ability to breathe air and live a semi-terrestrial existence. Pseudothelphusids reach their highest diversity in the rivers and streams that drain the highland areas of the cordilleras of Colombia and Ecuador. In contrast, trichodactylids are most abundant in the vast network of lowland rivers of South America that make up the Amazon, Orinoco, Paraguay, and Parana basins (Cumberlidge et al. 2009). Species diversity tends to be highest where vegetation cover is densest and water availability is highest, and there are fewer, or no species found in the more arid ecosystems and those with colder water temperatures. The pattern of the dominance of Pseudothelphusidae in Colombia, Venezuela, Ecuador, Peru, Mexico, Central America, and the Caribbean, and their absence in Bolivia, Uruguay, Paraguay, southern Argentina, and Chile probably reflects an origin somewhere in Colombia and Venezuela, from where they spread out north to Central America and Mexico, east to the Guyanas and Lesser Antilles, and south as far as Brazil and Peru (Rodríguez 1982, 1986).

Similarly the dominance of Trichodactylidae in the Amazon, Orinoco, Paraguay, and Parana River basins and their absence from Chile and from aquatic ecosystems at high altitudes in the Andes elsewhere in South America reflects their preference for an aquatic, rather than a semiterrestrial life (Rodríguez 1982, 1986). However, it is harder to explain the presence of trichodactylids in Central America, Mexico, and Trinidad in localities that lie well outside of the major river basins of the South American subcontinent (Rodríguez 1992).

Twenty-four species of neotropical freshwater crabs are found in caves (22 pseudothelphusids and two trichodactylids), and are either fully adapted to a life in caves, or are visitors to caves and have a distribution that also includes surface waters (Table 3). Twelve species of cave-dwelling freshwater crabs are from Mexico, five from Central America (Belize 1, Guatemala 3, and Costa Rica 1), two from the Caribbean (Cuba 1, Puerto Rico, and St. Croix 1), and five from South America (Colombia, 2 and Venezuela 3).

**Table 3.** Species of Neotropical freshwater crabs that are associated with caves, and the country where each species occurs. The family, subfamily/tribe for each species are also shown, together with its IUCN Red List conservation status. LC = Least Concern, EN = Endangered, DD = Data Deficient. For explanation of Red List categories see IUCN (2003). * = recently described species, not assessed yet for conservation status.

Family	Tribe	Species	Country	Cons. Status
Pseudothelphusidae	Strengerianini	*Neostrengeria charalensis*	Colombia	LC
Pseudothelphusidae	Strengerianini	*Neostrengeria sketi*	Colombia	DD
Pseudothelphusidae	Hypolobocerini	*Achlidon puntarenas*	Costa Rica	DD
Pseudothelphusidae	Epilobocerinae	*Epilobocera cubensis*	Cuba	LC
Pseudothelphusidae	Hypolobocerini	*Phrygiopilus acanthophallus*	Guatemala	DD
Pseudothelphusidae	Potamocarcinini	*Typhlopseudothelphusa juberthiei*	Guatemala	DD
Pseudothelphusidae	Potamocarcinini	*Zilchia falcata*	Guatemala	DD
Pseudothelphusidae	Potamocarcinini	*Typhlopseudothelphusa acanthochela*	Belize	DD
Trichodactylidae	Trichodactylinae	*Avotrichodactylus constrictus*	Mexico	LC
Trichodactylidae	Trichodactylinae	*Rodriguezia mensabak*	Mexico	DD
Pseudothelphusidae	Hypolobocerini	*Phrygiopilus montebelloensis*	Mexico	DD
Pseudothelphusidae	Hypolobocerini	*Phrygiopilus yoshibensis*	Mexico	DD
Pseudothelphusidae	Potamocarcinini	*Odontothelphusa monodontis*	Mexico	DD
Pseudothelphusidae	Potamocarcinini	*Potamocarcinus leptomelus*	Mexico	DD
Pseudothelphusidae	Pseudothelphusini	*Pseudothelphusa mexicana*	Mexico	DD
Pseudothelphusidae	Pseudothelphusini	*Tehuana complanata*	Mexico	DD
Pseudothelphusidae	Potamocarcinini	*Typhlopseudothelphusa hyba*	Mexico	DD
Pseudothelphusidae	Potamocarcinini	*Typhlopseudothelphusa mocinoi*	Mexico	EN
Pseudothelphusidae	Potamocarcinini	*Sylvathelphusa cavernicola*	Mexico	*
Pseudothelphusidae	Potamocarcinini	*Villalobosus lopezformenti*	Mexico	DD
Pseudothelphusidae	Epilobocerinae	*Epilobocera sinuatifrons*	Puerto Rico	LC
Pseudothelphusidae	Strengerianini	*Chaceus caecus*	Venezuela	DD
Pseudothelphusidae	Strengerianini	*Chaceus turikensis*	Venezuela	DD
Pseudothelphusidae	Pseudothelphusini	*Rodriguezus garmani*	Venezuela	LC

### Freshwater crab endemism (Table 4)

The majority of freshwater crab species found in Colombia (81%) are country endemics: 78 species of pseudothelphsids and 5 species of trichodactylids (Cumberlidge et al. 2009). The high degree of endemism in Colombia’s freshwater crab fauna at the species level (83 of 102 species, 81%) is also seen to a lesser extent at the genus level (9 of 21 genera, 43%), but not at the family level (neither of the two families are exclusively found in Colombia). Many of the Colombian endemics are found in the isolated mountain streams and in the middle stretches of rivers associated with rainforest (Campos 2005). Mexico’s freshwater crab fauna has the next highest number of country endemics (66 of 67 species, 97%) that is also seen at the genus level (13 of 17 genera, 76%), but not at the family level (neither of the two families are exclusively found in Mexico). Many of the Mexican endemics are found in the isolated mountain streams and in the middle stretches of rivers associated with the rainforests and caves (Villalobos and Alvarez 2010). All species of freshwater crabs found in Cuba and Hispaniola are island endemics.

**Table 4.** Countries that host endemic species of Neotropical freshwater crabs. First number indicates the number of species of Pseudothelphusidae (P), the second number indicates the same data for the Trichodactylidae (T).

Country	No. Endemic Sp. (P, T)
Colombia	78, 5
Mexico	62, 4
Venezuela	22, 0
Panama	12, 1
Ecuador	11, 0
Costa Rica	9, 0
Cuba	9, 0
Guatemala	9, 0
Brazil	7, 10
Peru	6, 3
El Salvador	3, 0
Nicaragua	3, 0
Belize	2, 0
Dominican Republic	2, 0
Guyana	2, 0
Haiti	1, 0
Honduras	1, 0
Suriname	1, 0
Trinidad	1, 0
Bolivia	0, 1

### Conservation status

The conservation status of the 298 species of freshwater crabs known from the Neotropical region in 2009 was evaluated against the IUCN (2003) Red List criteria (version 3.1) and is available on the Red List site (www.iucn.redlist.org). These results were summarized by Cumberlidge et al. (2009). The conservation status of species described since that work (Table 1) has not yet been assessed. Unfortunately, about half of all Neotropical freshwater crabs (148 species) are too poorly known to submit to the assessment protocols and are listed as Data Deficient, notably some 80% of the diverse Mexican fauna (Tables 5, 6). Of the remaining 150 species that could be assessed (belonging to 22 genera and 2 families) the majority (107 species) were found to be of Least Concern, and most of these species either live in rivers, marshy lowlands, or in mountain streams in the forested highlands (Cumberlidge et al. 2009). For the Pseudothelphusidae, 111 out of 251 species were assessed (140 species were data deficient), and of these, 72 species of Least Concern, 2 species were Critically Endangered, 3 species were Endangered Critically Endangered, 3 species were Endangered, and 33 species were Vulnerable (Tables 5, 6). For the Trichodactylidae, 39 out of 47 species were assessed (8 species were Data Deficient), and of these, 35 species were of Least Concern, one species was Endangered, and three were Vulnerable (Table 6). For both families combined, 42 of the 150 assessed species of Neotropical freshwater crabs (28%) were listed in one of three threatened categories, either as Critically Endangered (2 species), Endangered (4 species) or Vulnerable (36 species) (Table 7). One species was assessed as near threatened, and no species of Neotropical freshwater crabs was confirmed as Extinct or Extinct in the Wild. However, it should be noted that a species cannot be formally assessed as extinct until exhaustive surveys probing its disappearance have been carried out. Because conservation status could not be assigned to large numbers of Data Deficient species (148), the proportion of threatened species in Cumberlidge et al. (2009) (32%) is almost certainly an underestimation. This means that the number of threatened species could well increase should a Data Deficient species subsequently be assessed as threatened (Cumberlidge et al. 2009). Despite the large numbers of specimens available very little information is available on population levels and trends, except for a qualitative estimate (e.g., whether common or rare) based on the number of sites at which a species is present and its relative abundance at each site.

**Table 5.** Summary of the Red List assessments of the two families of Neotropical freshwater crabs based on data from Cumberlidge et al. (2009). Assessed = number of species that could be assessed using the IUCN Red List protocols; Thr. = total number of species assessed in one of the threatened categories (VU = Vulnerable, EN = Endangered, CR = Critically Endangered); LC = Least Concern, NT = Near Threatened, DD = Data Deficient. For explanation of Red List categories see IUCN (2003).

Family	Total Sp.	Assessed	Thr.	Thr. (%)	VU	EN	CR	LC	NT	DD
Pseudothelphusidae	251	111	38	34.2	33	3	2	72	1	140
Trichodactylidae	47	39	4	10.3	3	1	0	35	0	8
Total	298	150	42	28.3	36	4	2	107	1	148

**Table 6.** Summary of the countries in the Neotropical region that have threatened species of freshwater crabs based on data from Cumberlidge et al. (2009). Assessed = number of species that could be assessed using the IUCN Red List protocols; Thr. = total number of species assessed in one of the threatened categories (VU = Vulnerable, EN = Endangered, CR = Critically Endangered); LC = Least Concern, NT = Near Threatened, DD = Data Deficient.

Country	Total Sp.	Assessed	Thr.	Thr. (%)	VU	EN	CR	LC	NT	DD
Colombia	101	60	14	23.3	13	0	1	45	1	41
Venezuela	42	31	7	22.6	7	0	0	24	0	11
Mexico	63	19	6	31.6	2	3	1	13	0	44
Ecuador	27	19	5	36.8	5	0	0	12	0	8
Peru	25	21	3	14.3	3	0	0	0	0	4
El Salvador	8	6	2	33.3	2	0	0	4	0	2
Costa Rica	13	7	2	28.6	2	0	0	5	0	6
Brazil	45	38	2	10.5	1	1	0	34	0	7
Suriname	11	10	1	10.0	1	0	0	0	0	1
Honduras	6	6	1	33.3	1	0	0	4	0	0
Haiti	1	1	1	100.0	1	0	0	0	0	0
Guatemala	13	7	1	28.6	1	0	0	5	0	6
Dominican Rep.	2	2	1	100.0	1	0	0	0	0	0
Cuba	9	9	1	11.0	1	0	0	0	0	0

**Table 7.** Threatened species of Neotropical freshwater crabs. P = Pseudothelphusidae, T = Trichodactylidae. For explanation of Red list categories see IUCN (2003).

Family/Subfamily/Tribe	Taxon	IUCN Red List Category	Distribution
T. (Trichodactylinae)	*Trichodactylus crassus*	EN B1ab(iii)	Brazil
P. (Kingsleyini)	*Kingsleya siolii*	VU B1ab(iii); D2	Brazil, Suriname
P. (Kingsleyini)	*Fredius platyacanthus*	VU B1ab(iii); D2	Brazil, Venezuela
P. (Kingsleyini)	*Fredius stenolobus*	VU B1ab(iii); D2	Brazil, Venezuela
P. (Hypolobocerini)	*Hypolobocera alata*	VU B1ab(iii); D2	Colombia
P. (Hypolobocerini)	*Hypolobocera andagoensis*	VU B1ab(iii); D2	Colombia
P. (Hypolobocerini)	*Hypolobocera barbacensis*	VU B1ab(iii); D2	Colombia
P. (Hypolobocerini)	*Hypolobocera cajambrensis*	VU B1ab(iii); D2	Colombia
P. (Hypolobocerini)	*Hypolobocera rotundilobata*	VU B1ab(iii); D2	Colombia
P. (Hypolobocerini)	*Hypolobocera velezi*	VU B1ab(iii); D2	Colombia
P. (Hypolobocerini)	*Moritschus altaquerensis*	VU B1ab(iii); D2	Colombia
P. (Kingsleyini)	*Fredius granulatus*	VU B1ab(iii); D2	Colombia
P. (Strengerianini)	*Chaceus ibiricensis*	VU B1ab(iii)+2ab(iii); D2	Colombia
P. (Strengerianini)	*Strengeriana antioquensis*	CR B1ab(i,ii,iii,iv,v)+2ab(i,ii,iii,iv,v)	Colombia
T. (Dilocarcininae)	*Bottiella medemi*	VU B1ab(iii)+2ab(iii)	Colombia
P. (Hypolobocerini)	*Hypolobocera exuca*	VU B1ab(iii); D2	Colombia, Ecuador
P. (Hypolobocerini)	*Lindacatalina sumacensis*	VU B1ab(iii); D2	Colombia, Ecuador
T. (Dilocarcininae)	*Bottiella cucutensis*	VU B1ab(iii)+2ab(iii	Colombia, Venezuela
P. (Potamocarcini)	*Allacanthos pittieri*	VU B1ab(iii); D2	Costa Rica
P. (Potamocarcini)	*Ptychophallus tristani*	VU B1ab(iii)+2ab(iii); D2	Costa Rica
P. (Epilobocerinae	*Epilobocera wetherbeei*	VU B1ab(iii); D2	Dominican Republic
P. (Hypolobocerini)	*Hypolobocera delsolari*	VU B1ab(iii); D2	Ecuador
P. (Hypolobocerini)	*Hypolobocera rathbuni*	VU B1ab(iii); D2	Ecuador
P. (Hypolobocerini)	*Moritschus ecuadorensis*	VU B1ab(iii); D2	Ecuador
P. (Hypolobocerini)	*Elsalvadoria zurstrasseni*	VU B1ab(iii)+2ab(iii); D2	El Salvador
P. (Hypolobocerini)	*Raddaus mertensi*	VU D2	El Salvador, Guatemala, Honduras
P. (Hypolobocerini)	*Phrygiopilus acanthophallus*	VU B1ab(iii); D2	Guatemala
P. (Epilobocerinae)	*Epilobocera haytensis*	VU B1ab(iii)	Haiti, Dominican Rep.
P. (Potamocarcini)	*Potamocarcinus roatensis*	VU D2	Honduras
P. (Potamocarcini)	*Potamocarcinus hartmanni*	VU B1ab(ii)+2ab(ii); D2	Mexico
P. (Potamocarcini)	*Typhlopseudothelphusa mocinoi*	EN B1ab(iii)+2ab(iii)	Mexico
P. (Pseudothelphusini)	*Tehuana lamothei*	EN B1ab(ii,iii)+2ab(ii,iii)	Mexico
P. (Pseudothelphusini)	*Tehuana poglayenorum*	EN B1ab(ii,iii)+2ab(ii,iii)	Mexico
P. (Pseudothelphusini)	*Tehuana veracruzana*	CR B1ab(i,ii,iii,iv,v)+2ab(i,ii,iii,iv,v)	Mexico
T. (Trichodactylinae)	*Avotrichodactylus oaxensis*	VU B1ab(iii)+2ab(iii)	Mexico
P. (Hypolobocerini)	*Hypolobocera gracilignatha*	VU D2	Peru
P. (Hypolobocerini)	*Hypolobocera lamercedes*	VU B1ab(iii); D2	Peru
P. (Hypolobocerini)	*Hypolobocera peruviana*	VU D2	Peru
P. (Kingsleyini)	*Microthelphusa forcarti*	VU B1ab(iii); D2	Venezuela
P. (Kingsleyini)	*Neopseudothelphusa fossor*	VU B1ab(iii); D2	Venezuela
P. (Kingsleyini)	*Rodriguezus trujillensis*	VU B1ab(iii)+2ab(iii); D2	Venezuela

With only 42 out of 298 species of Neotropical freshwater crabs known in 2009 assessed as threatened with global extinction (Tables 5–7), the region’s largely endemic freshwater crab fauna (Table 4) does not appear at first sight to be in immediate trouble compared with other aquatic groups found in the same freshwater habitats (e.g., fish, molluscs, and dragonflies) (Collen et al. 2008). The 107 species of Neotropical freshwater crabs judged to be of Least Concern (35 species of trichodactylids, and 72 species of pseudothelphusids) have a wide distribution in rivers, lakes, highland streams and lowland wetlands, and appear to be tolerant of changes in land use that affect aquatic ecosystems.

Threats to Neotropical freshwater crabs include habitat destruction driven by increasing agriculture, the demands of increasing industrial development, and the alteration of fast flowing rivers for the creation of hydroelectric power (Cumberlidge et al. 2009). Even species assessed as Least Concern could suffer a catastrophic decline should there be abrupt changes in land development, hydrology, or pesticide-use regimes. Species with a narrow distribution are vulnerable to extreme population fragmentation and could suffer a rapid decline and even extinction in a relatively short time should dramatic changes in land-use affect their habitat. The 148 species of Neotropical freshwater crabs judged to be Data Deficient are mostly rare species, and their conservation status needs to be re-evaluated once more information comes to light. It is hoped that prioritizing species for conservation action through the Red List assessment process will lead to the development of conservation recovery plans for threatened species in the future.

## Conclusions

This study confirms that freshwater crabs are completely absent from all parts of the Nearctic region (even in the neighboring warmer parts of northern Mexico and southern Florida, USA), and that the northern limits of their distribution coincide with the Nearctic/ Neotropical boundary in Mexico and the presence of arid regions lacking permanent freshwaters (i.e., the Sonoran and Chihuahuan Deserts). Species richness of freshwater crabs increases south of this boundary in Mexico and reaches a peak in the States of Veracruz, Tabasco, Oaxaca, and Chiapas. The uneven pattern of species-richness – high in Mexico, low in Central America, and high in Colombia – is difficult to explain by simply invoking an even spread of species out of a center of origin somewhere in Colombia–Venezuela (Rodríguez 1982, 1992), and further studies are necessary. The relatively low species richness of freshwater crabs in Brazil is unexpected given its vast area and rich biodiversity, and may reflect the dominance of trichodactylids and a general paucity of species-rich pseudothelphusids in the Brazilian fauna. The abundance of freshwater crabs in Colombia, Ecuador, and Peru coincides with a freshwater habitat species diversity hotspot (that is defined by overlapping distributions of stenotopic species based on combined data from vertebrates and invertebrates (Collen et al. 2008)). All countries in the Neotropical region are still in a phase of exploration and steep rises in species numbers of pseudothelphusids are expected once more surveys have been completed.

The Neotropics are the second most diverse region in the world for freshwater crabs, and there are 42 species from 14 countries within this region that may be threatened with extinction. The finding that 34% of pseudothelphusids and 10% of trichodactylids are threatened, and that many others are poorly known, can be used to develop a conservation strategy for these species, and supply a focus for future research efforts (Cumberlidge et al. 2009). Although some species of Neotropical freshwater crabs have been quite well studied a clear majority are known only from either the type locality or from just a few localities, and in these cases further collections are necessary to ascertain their actual distributions. The restricted range of many species from the Neotropics, together with the on-going human induced loss of habitat in many parts of the region, are a cause for concern for the long-term security of elements of this fauna. Conservation activities should therefore be aimed primarily at preserving the integrity of sites and habitats while at the same time closely monitoring key populations. Significant areas of the Neotropics still remain insufficiently explored, and the focus of new efforts should be on new species discovery through increased collection in remote areas and the refinement of taxonomic skills. These efforts should also focus on conserving threatened species, and on seeking out data deficient species. Gathering current data on distribution, natural history, population trends, threats, and endemism of the Neotropical region’s highly diverse freshwater crabs will enable the updating of the IUCN Red Lists, and the development of conservation strategies for this understudied diverse and potentially highly threatened fauna.
